# Post-translational modification of P_**II**_ signal transduction proteins

**DOI:** 10.3389/fmicb.2014.00763

**Published:** 2015-01-06

**Authors:** Mike Merrick

**Affiliations:** Department of Molecular Microbiology, John Innes CentreNorwich, UK

**Keywords:** p_II_ protein, post-translational modification, uridylylation, adenylylation, phosphorylation

## Abstract

The P_II_ proteins constitute one of the most widely distributed families of signal transduction proteins in nature. They are pivotal players in the control of nitrogen metabolism in bacteria and archaea, and are also found in the plastids of plants. Quite remarkably P_II_ proteins control the activities of a diverse range of enzymes, transcription factors and membrane transport proteins, and in all known cases they achieve their regulatory effect by direct interaction with their target. P_II_ proteins in the Proteobacteria and the Actinobacteria are subject to post-translational modification by uridylylation or adenylylation respectively, whilst in some Cyanobacteria they can be modified by phosphorylation. In all these cases the protein’s modification state is influenced by the cellular nitrogen status and is thought to regulate its activity. However, in many organisms there is no evidence for modification of P_II_ proteins and indeed the ability of these proteins to respond to the cellular nitrogen status is fundamentally independent of post-translational modification. In this review we explore the role of post-translational modification in P_II_ proteins in the light of recent studies.

## THE P_II_ PROTEIN FAMILY

P_II_ proteins were first identified by B. M. Shapiro in the late 1960s when he was studying control of the activity of *Escherichia coli* glutamine synthetase (GS) by adenylylation/deadenylylation (Shapiro, 1969). Subsequent studies by Stadtman and co-workers characterized two proteins: P_I_ which was responsible for the adenylylation/deadenylylation of GS, and P_II_ which modulated these activities (Brown et al., 1971). Furthermore they observed that P_II_ itself existed in two forms, the interconversion of which appeared to involve the covalent attachment of a uridine derivative to P_II_. In due course it was shown that P_II_ was encoded by *glnB*, that uridylylation occurred on residue Tyr51 in the T-loop of the protein, and that both uridylylation and deuridylylation were effected by the enzymatic activity of GlnD (Arcondéguy et al., 2001; Huergo et al., 2013).

**FIGURE 1:**
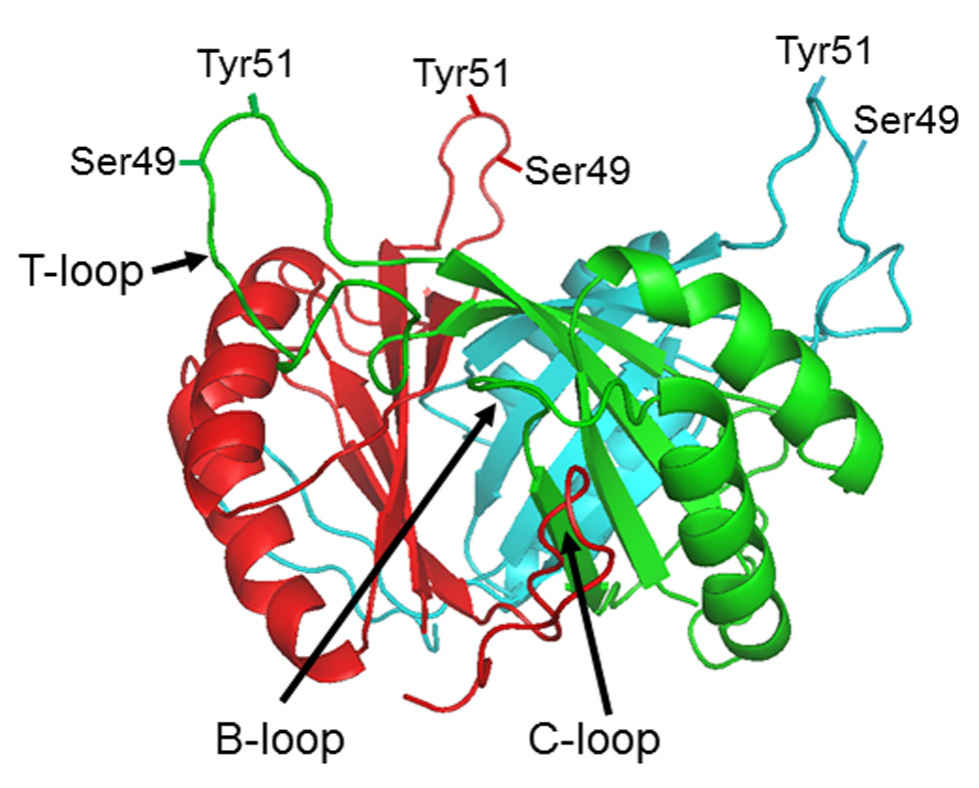
**P_II_ protein structure**. Side view of the *Escherichia coli* GlnB trimer (PDB: 2P_II_) in the absence of ligands showing the positions of the B, C, and T loops and the residues within the T-loop (Ser49 and Tyr51) which can be subject to post-translational modification in different organisms.

Extensive genomic research has shown that P_II_ proteins are extremely widespread amongst bacteria, archaea, and plants (Sant’Anna et al., 2009). They are, however, not found in fungi or animals. Their presence in plants is considered to be a consequence of the cyanobacterial origin of the plastid and the protein, whilst encoded in the nucleus, is expressed in the chloroplast (Chellamuthu et al., 2013). Bacteria and archaea often encode multiple P_II_ proteins, e.g., proteobacteria typically encode two, designated GlnB and GlnK. However, cyanobacteria and plants usually encode just a single copy. All P_II_ proteins show a very high level of sequence conservation and protein crystallography studies of P_II_ proteins from bacteria, archaea, and plants indicates that their tertiary structure is also highly conserved (**Figure 1**). They are homotrimers with a core 12–13 kDal subunit. The trimer forms a compact cylindrical-shaped molecule from which three long exposed loops (the T-loops) protrude. Two smaller loops, the B- and C-loops, are located at the interface between adjacent subunits of the trimer such that the T- and B-loops of one monomer and the C-loop of the adjacent monomer form an inter-subunit cleft that constitutes a ligand binding site. Although there is now extensive structural data on P_II_ proteins from a range of organisms no structures have been solved for uridylylated or phosphorylated forms (Huergo et al., 2013).

Whilst originally identified as regulating the activity of the GS adenylyltransferase in *E. coli,* P_II_ proteins are now known to control the activities of a very diverse range of enzymes, a large number of transcription factors and some membrane transport proteins. In all known cases they achieve their regulatory effect by direct interaction with their target and in the majority of cases studied to date that interaction involves the T-loops of the P_II_ protein.

The recognition that the T-loops are potentially very flexible and would be able to adopt a variety of structures together with the knowledge that uridylylation of the protein in proteobacteria occurs within the T-loop, suggested early on that post-translational modification could play a key role in facilitating the regulatory activity of P_II_ proteins. However, these proteins are able to bind both ATP/ADP and 2-oxoglutarate (2-OG) and biochemical and crystallographic studies indicate that these effectors alone can have major effects on T-loop structure and on the interaction of P_II_ proteins with many of their targets (Conroy et al., 2007; Jiang and Ninfa, 2007, 2009; Truan et al., 2010). ATP and ADP compete for binding to the same site in the inter-subunit cleft but 2-OG can only bind in the presence of Mg-ATP and ADP does not support 2-OG binding. The ability of P_II_ proteins to bind 2-OG allows them to respond to aspects of the cellular nitrogen and carbon status whilst it has been proposed that the competitive binding of ATP and ADP also makes P_II_ proteins potential sensors of the adenylylate energy charge.

The factors influencing effector binding and the physiological influences on their interactions with P_II_ are presently a matter of debate (Zeth et al., 2014). Changes of the effector pools *in vivo* under different physiological conditions have been studied in the case of the interaction of GlnK and AmtB in *E. coli* (Radchenko et al., 2010; Radchenko and Merrick, 2011). When the intracellular nitrogen status is high 2-OG levels in the cell are low and ADP is found bound to GlnK within the GlnK–AmtB complex (Conroy et al., 2007; Radchenko et al., 2013). The binding of ADP leads to concomitant change in the GlnK T-loop to form a structure in which the apex of the loop projects 28Å above the core of the protein facilitating complex formation with AmtB (Conroy et al., 2007). Conversely when the intracellular nitrogen status is low, 2-OG levels in the cell are high (Radchenko et al., 2013). Under these conditions GlnK is expected to bind 2-OG and Mg-ATP, the T-loops are relatively unstructured and complex formation is not promoted (Truan et al., 2010, 2014).

The switch between the ATP and ADP bound forms of GlnK has been ascribed by Radchenko et al. (2013) to an ATPase activity of the protein that is only manifest in low 2-OG. Other authors have proposed that the nucleotide occupancy of the effector binding site is solely a reflection of the cellular ATP/ADP ratio (Jiang and Ninfa, 2007, 2009; Fokina et al., 2011; Zeth et al., 2014). Radchenko et al. (2013) propose that a drop in the intracellular 2-OG pool promotes hydrolysis of ATP to ADP leading to a rearrangement of residues in the binding pocket, most notably Gln39 and K58, and a concomitant change in the T-loop structure. Such a model is consistent with the observed GlnK–AmtB and P_II_–PipX structures and the mode of action of the P_II_ proteins in both these cases (see later). However, it is not consistent with *in vitro* studies of P_II_–*N*-acetylglutamate kinase (NAGK) complex formation which indicate that this interaction is promoted by ATP alone and is inhibited by 2-OG (Fokina et al., 2011; Zeth et al., 2014).

In summary, changes in T-loop structure in response to changes in cellular N status as reflected by the 2-OG pool, or possibly in response to changes in the cellular ATP/ADP ratio, are sufficient to explain the ability of P_II_ proteins to interact with their cognate targets. Consequently the role of post-translational modification of P_II_ proteins is a facet of P_II_ biology that needs to be re-evaluated and studied in much more detail.

## URIDYLYLATION AND ADENYLYLATION OF P_II_ PROTEINS

P_II_ uridylylation and deuridylylation are carried out by a single enzyme, encoded by *glnD,* that has been characterized from a number of proteobacteria. GlnD proteins have a molecular mass of about 100 kDal and contain a conserved nucleotidyltransferase superfamily motif. They have at least four domains of which the N-terminal domain encodes the uridylyltransferase (UTase) activity and the adjacent HD domain encodes the uridylyl-removing (UR) activity (Jiang et al., 1998; Zhang et al., 2010): hence, the two activities are not thought to share an active site. GlnD has a single glutamine-binding site and its activity is regulated by the intracellular glutamine level such that UTase activity predominates in low glutamine and UR activity is stimulated by high glutamine levels. P_II_ is the only known substrate for GlnD and the regulation of GlnD activity by glutamine means that in organisms where P_II_ is subject to uridylylation both the 2-OG and the glutamine pools influence P_II_ activity. The metabolic links between 2-OG and glutamine mean that usually the levels of these two effectors change in a reciprocal manner but there may be physiological conditions where the two are at least partially uncoupled.

**FIGURE 2:**
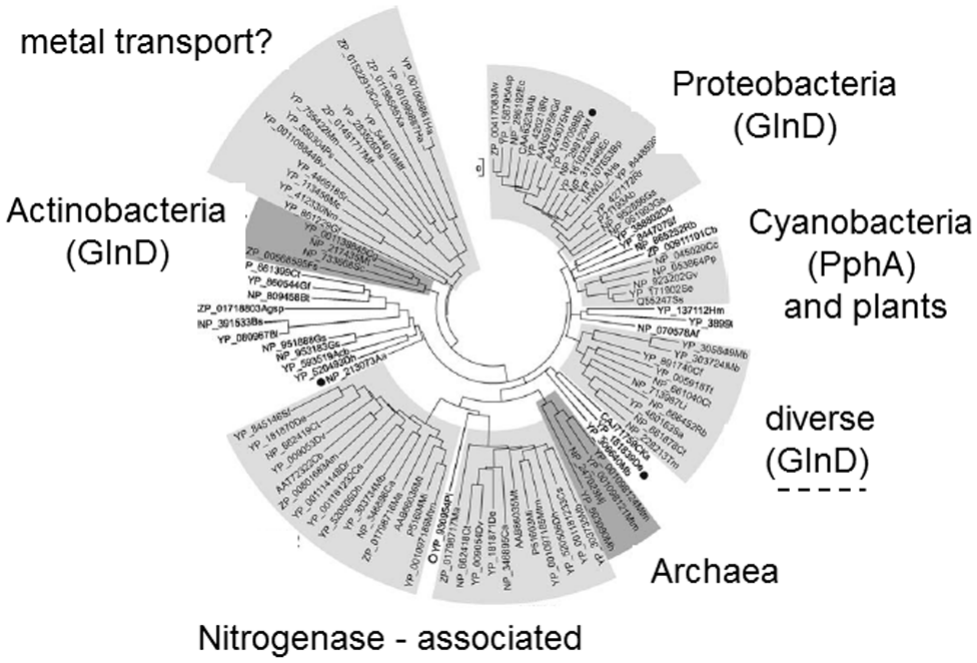
**Phylogenetic tree of P_II_ proteins showing those groups in which T-loop modification occurs**. Phylogenetic analysis of the P_II_ protein family (Sant’Anna et al., 2009) identified at least seven distinct groups four of which are associated with specific taxonomic groups of organisms. Two groups appear to be associated with specific processes in taxonomically diverse organisms: one group control nitrogenase in certain diazotrophs (Leigh and Dodsworth, 2007) and the other group is often genetically linked to metal transport genes. Post-translational modification has only been described in four groups within the tree. Uridylylation (mediated by GlnD) is found in the proteobacteria and some “diverse” bacteria. Adenylylation (also mediated by GlnD) is found in the actinobacteria. Phosphorylation (associated with the presence of the phosphatase PphA) is found in some cyanobacteria but not in plants.

The *glnD* gene is found ubiquitously in the proteobacteria and the actinobacteria, and sporadically in a few other diverse genera (Huergo et al., 2013). In the actinobacteria *glnD* is part of the *amtB, glnK, glnD* operon, whereas in the proteobacteria it is usually encoded elsewhere in the genome. Furthermore, studies of P_II_ modification in two actinobacteria, *Streptomyces coelicolor* and *Corynebacterium glutamicum*, found that in both these organisms the activity of GlnD is to adenylylate the single P_II_ protein, GlnK, rather than to uridylylate it (Hesketh et al., 2002; Strosser et al., 2004). The modification takes place on the equivalent Tyr51 residue of the T-loop and the exact basis for transfer of an adenyl rather than an uridyl group has not been determined. Hence, it is clear that within the P_II_ family post-translational modification by uridylylation/adenylylation is relatively restricted (**Figure 2**), and as with the P_II_ proteins (Sant’Anna et al., 2009) the distribution of *glnD* also suggests that horizontal gene transfer may have played a part in its present distribution.

## PHOSPHORYLATION OF P_II_ PROTEINS

An alternative form of post-translational modification of P_II_ occurs in the cyanobacteria. In this case the T-loop is subject to phosphorylation on residue Ser49 and, as with uridylylation and adenylylation, the modification occurs in response to nitrogen limitation. T-loop phosphorylation has been observed in *Synechococcus elongatus* and in *Synechocystis* but despite conservation of residue Ser49 in the T-loop phosphorylation is not found in other cyanobacteria such as *Prochlorococcus* and *Anabaena* (Forchhammer et al., 2004). Furthermore, there is no evidence for post-translational modification of P_II_ in plants (Smith et al., 2004). A completely novel modification, namely nitration of Tyr51, has been reported for *Anabaena* (Zhang et al., 2007).

The mechanism of P_II_ modification in cyanobacteria is also not fully understood. Dephosphorylation is driven by a specific phosphatase, PphA, the activity of which is inhibited by 2-OG in concert with Mg-ATP (Irmler and Forchhammer, 2001; Ruppert et al., 2002). Hence, 2-OG plays a key role as an effector, both by binding to P_II_ in conditions of N-limitation and also by inhibiting the phosphatase and ensuring that P_II_ remains phosphorylated in this situation. However, despite considerable efforts, the kinase responsible for phosphorylation of P_II_ has yet to be identified and consequently the factors regulating its activity are also still unknown (Chellamuthu et al., 2013). Potentially the kinase could offer another signal transduction route into the system, for example if glutamine was to inhibit the kinase in an analogous mode to the inhibition by glutamine of GlnD UTase activity, but at present such hypotheses await discovery of the kinase.

## THE ROLE OF P_II_ MODIFICATION

An assessment of the various taxonomic groups of P_II_ proteins identified by Sant’Anna et al. (2009) makes it clear that many members of the family are probably not subject to any form of post-translational modification (**Figure 2**). There are to date no reports of P_II_ modification in archaea, nor in the Firmicutes such as *Bacillus subtilis*. The novel group of P_II_ proteins that function specifically to regulate activity of the nitrogenase enzyme in a variety of bacteria have also not been found to be controlled by post-translational modification and, as discussed above, modification is scattered amongst groups such as the cyanobacteria. So it would appear that P_II_ modification has arisen more than once in evolution and that many P_II_ proteins function effectively without such modification.

To assess the role of P_II_ modification in those organisms where it occurs it is necessary to examine model systems in which the interaction between a P_II_ protein and its target has been characterized in some detail, preferably both biochemically and structurally.

### GlnK–AmtB

Regulation of the ammonium transporter AmtB by the P_II_ protein GlnK is widespread in both bacteria and archaea. The structural genes for these proteins are invariably linked in a single operon (*glnK amtB* or *amtB glnK*) and it has been suggested that this is the evolutionary origin of the P_II_ protein family (Thomas et al., 2000; Sant’Anna et al., 2009). The interaction of GlnK with AmtB has been studied in considerable detail both *in vivo* and *in vitro* and the structure of the complex from *E. coli* has been solved.

In N-limited conditions GlnK is fully uridylylated and free in the cytoplasm with Mg-ATP and 2-OG bound in the effector binding pocket as described earlier. An increase in the cellular N status leads to deuridylylation of GlnK and its sequestration to the inner membrane by AmtB (Radchenko et al., 2010). The crystal structure of the *E. coli* AmtB–GlnK complex shows that GlnK interacts with AmtB almost exclusively via the T-loop, the tip of which inserts deeply into the cytoplasmic pore exit of AmtB, thus blocking ammonia conduction into the cell (Conroy et al., 2007). The AmtB-bound GlnK has ADP bound in all three effector binding pockets. It is clear from the structure of the complex that uridylylation of GlnK will prevent complex formation and that deuridylylation is therefore a prerequisite for binding to GlnK. However, studies using a Tyr51Ala variant of GlnK that cannot be uridylylated have shown that this protein still responds to changes in cellular nitrogen status in a manner almost identical to the wild-type GlnK (Radchenko et al., 2014). As might be expected, when cells are subject to an ammonium shock the Tyr51Ala variant binds to AmtB slightly faster than the wild-type, presumably because the protein does not have to undergo deuridylylation prior to complex formation. Hence regulation of ammonia flux through AmtB by GlnK is not dependent on a functional T-loop modification and this is consistent with the fact that the GlnK–AmtB system is found in many organisms that do not exhibit post-translational modification of P_II_.

### P_II_–PipX

This complex has been studied in the cyanobacterium *S. elongatus* where PipX is a co-activator of the transcription factor NtcA. P_II_ interacts with PipX to antagonize PipX–NtcA complex formation (Espinosa et al., 2006). In N-limited conditions de-phosphorylated P_II_ binds to PipX thereby freeing NtcA to activate transcription of genes required for growth in N-limitation. Complex formation between P_II_ and PipX is strongly reduced by ATP in concert with 2-OG, conditions that occur under N-sufficiency (Llacer et al., 2010). The complex has a 3:3 stoichiometry such that one trimer of P_II_ binds three molecules of PipX that are “caged” between the extended T-loops of the P_II_ trimer (Llacer et al., 2010; Zhao et al., 2010). The T-loop conformation is similar to that seen in the GlnK–AmtB complex and consistent with this ADP has been shown to increase the affinity of P_II_ for PipX although no bound nucleotide was present in the structure of the complex. Residue Ser49 of the T-loop in P_II_ is positioned such that phosphorylation of Ser49 would not disrupt the complex and studies with a Ser49Glu variant that mimics phosphorylation confirm this (Llacer et al., 2010). Hence control of P_II_–PipX association and dissociation appears to be independent of post-translational modification of P_II_.

### P_II_–NAGK

*N*-acetylglutamate kinase is regulated by interaction with P_II_ in both cyanobacteria and plants (Burillo et al., 2004; Heinrich et al., 2004). NAGK is a key enzyme in arginine biosynthesis and arginine is used as a nitrogen store in these organisms. In conditions of N-sufficiency P_II_ binds to NAGK and enhances the enzyme’s catalytic activity thereby increasing cellular arginine levels and allowing arginine to be used for nitrogen storage in the form of cyanophycin (Forchhammer, 2008).

Structures of the complex have been solved for both *S. elongatus* and *Arabidopsis thaliana* and both structures are very similar (Llacer et al., 2007; Mizuno et al., 2007). In each case a hexamer of NAGK is sandwiched between two P_II_ trimers and the T-loops constitute a major interface with NAGK. The *S. elongatus* complex had no bound nucleotide but the very similar *A. thaliana* complex had bound Mg-ATP. In *S. elongatus* ADP has been reported to inhibit P_II_–NAGK complex formation (Maheswaran et al., 2004).

A Ser49Glu variant of P_II_ is unable to form a complex with NAGK (Heinrich et al., 2004) suggesting that phosphorylation inhibits the interaction and the structure of the complex clarifies how this occurs. Upon phosphorylation, one donor hydrogen bond formed with NAGK by the Ser49 OH group is lost. Furthermore, the bulkiness and negative charge of the phosphate triggers a steric and electrostatic clash with NAGK binding. However, it would appear that P_II_ phosphorylation is likely to occur after dissociation from NAGK and that, as with the other characterized P_II_ complexes, modification does not directly control complex formation and dissociation. It should also be noted that *A. thaliana* P_II_ is not subject to phosphorylation suggesting that, at least in this plant, NAGK regulation by P_II_ can function in the absence of post-translational modification.

### GlnZ–DraG

In the diazotrophic proteobacterium *Azospirillum brasilense* the NifH subunit of nitrogenase is subject to post-translational modification by ADP-ribosylation and this modification is mediated by an ADP-ribosyltransferase (DraT) and an ADP-ribosylhydrolase (DraG; Huergo et al., 2012). The antagonistic activities of both DraT and DraG are regulated by P_II_ proteins; DraT by GlnB and DraG by GlnZ. In N-limited conditions both GlnB and GlnZ are uridylylated and do not interact with DraT and DraG. However, in N-sufficient conditions both P_II_ proteins undergo deuridylylation and then interact with their respective target proteins. The structures of DraT and of the GlnB–DraT complex have yet to be solved so the role of T-loop modification in regulating DraT activity is not known. However, the structures of both DraG and of the GlnZ–DraG complex have been solved (Berthold et al., 2009; Li et al., 2009; Rajendran et al., 2011). The complex is unique amongst those P_II_ complexes for which structural information is presently available because the interface between the two proteins does not involve the T-loops. Hence GlnZ uridylylation will not apparently have a major influence on its interaction with DraG. Structural modeling indicates that DraG is inactivated when bound to GlnZ due to steric hindrance of the DraG active site (Rajendran et al., 2011).

## CONCLUSION

Whilst post-translational modification has been recognized as a key feature of P_II_ protein biology since its recognition in *E. coli* in the early 1970s, subsequent studies have determined that it is not a universal feature of this large protein family. In the case of uridylylation mediated by GlnD in the proteobacteria (and probably the equivalent adenylylation in archaea) this modification appears to serve to allow integration of sensing of the glutamine pool, through the regulation of GlnD activity by glutamine, with sensing of the 2-oxoglutarate pool by direct binding to P_II_. In those cases where detailed biochemical and structural studies are available post-translational modification does not appear to be essential for regulation of complex formation, at least in the physiological conditions studied. However, it may influence the dynamics of the process.

The phosphorylation of P_II_ proteins seen in some cyanobacteria also has the potential to facilitate additional sensory input through regulation of the P_II_-specific kinase but as the kinase has yet to be identified and characterized this concept remains hypothetical at present. Where cyanobacterial P_II_ systems involving phosphorylation have been characterized post-translational modification again does not appear to be essential for regulation of complex formation, and this is supported by the fact that P_II_ phosphorylation is not ubiquitous in the cyanobacteria (Forchhammer et al., 2004).

In summary, from the studies undertaken to date there appears to be no unifying role for post-translational modification of P_II_ proteins. Interaction of P_II_ proteins with their targets is predominantly controlled by effector binding (MgATP, ADP, and 2-OG) and consequent changes in T-loop conformation (Truan et al., 2010; Radchenko and Merrick, 2011; Radchenko et al., 2013). In a number of cases post-translational modification appears to have the potential to operate as a check-point by providing another signal transduction input that has to be accommodated before complex formation between P_II_ and at least some of its targets can proceed.

There is clearly a need for much more information both with respect to studies of many more P_II_ interactions and in a more varied range of physiological conditions. Both types of study may reveal further important roles for P_II_ modification. It is also the case that the majority of studies to date have been of steady state situations and there is a definite need for more studies of P_II_ behavior under conditions of physiological transition because these could well be the situations where the influence of P_II_ modifications are most apparent.

## Conflict of Interest Statement

The author declares that the research was conducted in the absence of any commercial or financial relationships that could be construed as a potential conflict of interest.

## References

[B1] Arcondéguy T. Jack R. Merrick M. (2001). PII signal transduction proteins: pivotal players in microbial nitrogen control. *Microbiol. Mol. Biol. Rev.* 65 80–105 10.1128/MMBR.65.1.80-105.2001 11238986 PMC99019

[B2] Berthold C. L. Wang H. Nordlund S. Hogbom M. (2009). Mechanism of ADP-ribosylation removal revealed by the structure and ligand complexes of the dimanganese mono-ADP-ribosylhydrolase DraG. *Proc. Natl. Acad. Sci. U.S.A.* 106 14247–14252 10.1073/pnas.0905906106 19706507 PMC2732831

[B3] Brown M. S. Segal A. Stadtman E. R. (1971). Modulation of glutamine synthetase adenylylation and deadenylylation is mediated by metabolic transformation of the PII regulatory protein. *Proc. Natl. Acad. Sci. U.S.A.* 68 2949–2953 10.1073/pnas.68.12.2949 4399832 PMC389567

[B4] Burillo S. Luque I. Fuentes I. Contreras A. (2004). Interactions between the nitrogen signal transduction protein PII and N-acetyl glutamate kinase in organisms that perform oxygenic photosynthesis. *J. Bacteriol.* 186 3346–3354 10.1128/JB.186.11.3346-3354.2004 15150219 PMC415743

[B5] Chellamuthu V. R. Alva V. Forchhammer K. (2013). From cyanobacteria to plants: conservation of PII functions during plastid evolution. *Planta* 237 451–462 10.1007/s00425-012-1801-0 23192387

[B6] Conroy M. J. Durand A. Lupo D. Li X. D. Bullough P. A. Winkler F. K. (2007). The crystal structure of the *Escherichia coli* AmtB-GlnK complex reveals how GlnK regulates the ammonia channel. *Proc. Natl. Acad. Sci. U.S.A.* 104 1213–1218 10.1073/pnas.0610348104 17220269 PMC1783118

[B7] Espinosa J. Forchhammer K. Burillo S. Contreras A. (2006). Interaction network in cyanobacterial nitrogen regulation: pipX, a protein that interacts in a 2-oxoglutarate dependent manner with PII and NtcA. *Mol. Microbiol.* 61 457–469 10.1111/j.1365-2958.2006.05231.x 16796668

[B8] Fokina O. Herrmann C. Forchhammer K. (2011). Signal-transduction protein PII from *Synechococcus elongatus* PCC 7942 senses low adenylate energy charge in vitro. *Biochem. J.* 440 147–156 10.1042/BJ20110536 21774788

[B9] Forchhammer K. (2008). PII signal transducers: novel functional and structural insights. *Trends Microbiol.* 16 65–72 10.1016/j.tim.2007.11.004 18182294

[B10] Forchhammer K. Irmler A. Kloft N. Ruppert U. (2004). P signalling in unicellular cyanobacteria: analysis of redox-signals and energy charge. *Physiol. Plant.* 120 51–56 10.1111/j.0031-9317.2004.0218.x 15032876

[B11] Heinrich A. Maheswaran M. Ruppert U. Forchhammer K. (2004). The *Synechococcus elongatus* P signal transduction protein controls arginine synthesis by complex formation with *N*-acetyl-l-glutamate kinase. *Mol. Microbiol.* 52 1303–1314 10.1111/j.1365-2958.2004.04058.x 15165234

[B12] Hesketh A. Fink D. Gust B. Rexer H. U. Scheel B. Chater K. (2002). The GlnD and GlnK homologues of *Streptomyces coelicolor* A3(2) are functionally dissimilar to their nitrogen regulatory system counterparts from enteric bacteria. *Mol. Microbiol.* 46 319–330 10.1046/j.1365-2958.2002.03149.x 12406211

[B13] Huergo L. F. Chandra G. Merrick M. (2013). PII signal transduction proteins: nitrogen regulation and beyond. *FEMS Microbiol. Rev.* 37 251–283 10.1111/j.1574-6976.2012.00351.x 22861350

[B14] Huergo L. F. Pedrosa F. O. Muller-Santos M. Chubatsu L. S. Monteiro R. A. Merrick M. (2012). PII signal transduction proteins: pivotal players in post-translational control of nitrogenase activity. *Microbiology* 158 176–190 10.1099/mic.0.049783-0 22210804

[B15] Irmler A. Forchhammer K. (2001). A PP2C-type phosphatase dephosphorylates the PII signaling protein in the cyanobacterium *Synechocystis* PCC 6803. *Proc. Natl. Acad. Sci. U.S.A.* 98 12978–12983 10.1073/pnas.231254998 11687619 PMC60810

[B16] Jiang P. Ninfa A. J. (2007). *Escherichia coli* PII signal transduction protein controlling nitrogen assimilation acts as a sensor of adenylate energy charge in vitro. *Biochemistry* 46 12979–12996 10.1021/bi701062t 17939683

[B17] Jiang P. Ninfa A. J. (2009). Sensation and signaling of alpha-ketoglutarate and adenylylate energy charge by the *Escherichia coli* PII signal transduction protein require cooperation of the three ligand-binding sites within the PII trimer. *Biochemistry* 48 11522–11531 10.1021/bi9011594 19877670 PMC2786245

[B18] Jiang P. Peliska J. A. Ninfa A. J. (1998). Enzymological characterization of the signal-transducing uridylyltransferase/uridylyl-removing enzyme (EC 2.7.7.59) of *Escherichia coli* and its interaction with the PII protein. *Biochemistry* 37 12782–12794 10.1021/bi980667m 9737855

[B19] Leigh J. A. Dodsworth J. A. (2007). Nitrogen regulation in bacteria and archaea. *Annu. Rev. Microbiol.* 61 349–377 10.1146/annurev.micro.61.080706.093409 17506680

[B20] Li X. D. Huergo L. F. Gasperina A. Pedrosa F. O. Merrick M. Winkler F. K. (2009). Crystal structure of dinitrogenase reductase activating glycohydrolase (DRAG) reveals conservation in the ADP-ribosylhydrolase fold and specific features in the ADP-ribose-binding pocket. *J. Mol. Biol.* 390 737–746 10.1016/j.jmb.2009.05.031 19477184

[B21] Llacer J. L. Contreras A. Forchhammer K. Marco-Marin C. Gil-Ortiz F. Maldonado R. (2007). The crystal structure of the complex of PII and acetylglutamate kinase reveals how PII controls the storage of nitrogen as arginine. *Proc. Natl. Acad. Sci. U.S.A.* 104 17644–17649 10.1073/pnas.0705987104 17959776 PMC2077032

[B22] Llacer J. L. Espinosa J. Castells M. A. Contreras A. Forchhammer K. Rubio V. (2010). Structural basis for the regulation of NtcA-dependent transcription by proteins PipX and PII. *Proc. Natl. Acad. Sci. U.S.A.* 107 15397–15402 10.1073/pnas.1007015107 20716687 PMC2932567

[B23] Maheswaran M. Urbanke C. Forchhammer K. (2004). Complex formation and catalytic activation by the PII signaling protein of *N*-acetyl-L-glutamate kinase from *Synechococcus elongatus* strain PCC 7942. *J. Biol. Chem.* 279 55202–55210 10.1074/jbc.M410971200 15502156

[B24] Mizuno Y. Moorhead G. B. Ng K. K. (2007). Structural basis for the regulation of *N*-acetylglutamate kinase by PII in *Arabidopsis thaliana*. *J. Biol. Chem.* 282 35733–35740 10.1074/jbc.M707127200 17913711

[B25] Radchenko M. Merrick M. (2011). The role of effector molecules in signal transduction by PII proteins. *Biochem. Soc. Trans.* 39 189–194 10.1042/BST0390189 21265771

[B26] Radchenko M. V. Thornton J. Merrick M. (2010). Control of AmtB-GlnK complex formation by intracellular levels of ATP, ADP and 2-oxoglutarate. *J. Biol. Chem.* 285 31037–31045 10.1074/jbc.M110.153908 20639578 PMC2945594

[B27] Radchenko M. V. Thornton J. Merrick M. (2013). P(II) signal transduction proteins are ATPases whose activity is regulated by 2-oxoglutarate. *Proc. Natl. Acad. Sci. U.S.A.* 110 12948–12953 10.1073/pnas.1304386110 23818625 PMC3740892

[B28] Radchenko M. V. Thornton J. Merrick M. (2014). Association and dissociation of the GlnK-AmtB complex in response to cellular nitrogen status can occur in the absence of GlnK post-translational modification. *Front. Microbiol.* 5:731 10.3389/fmicb.2014.00731 PMC4274968 25566239

[B29] Rajendran C. Gerhardt E. C. Bjelic S. Gasperina A. Scarduelli M. Pedrosa F. O. (2011). Crystal structure of the GlnZ-DraG complex reveals a different form of PII-target interaction. *Proc. Natl. Acad. Sci. U.S.A.* 108 18972–18976 10.1073/pnas.1108038108 22074780 PMC3223478

[B30] Ruppert U. Irmler A. Kloft N. Forchhammer K. (2002). The novel protein phosphatase PphA from *Synechocyctis* PCC 6803 controls dephosphorylation of the signalling protein PII. *Mol. Microbiol.* 44 855–864 10.1046/j.1365-2958.2002.02927.x 11994164

[B31] Sant’Anna F. H. Trentini D. B. de Souto W. S. Cecagno R. da Silva S. C. Schrank I. S. (2009). The PII superfamily revised: a novel group and evolutionary insights. *J. Mol. Evol.* 68 322–336 10.1007/s00239-009-9209-6 19296042

[B32] Shapiro B. M. (1969). The glutamine synthetase deadenylylating enzyme system from *Escherichia coli*. Resolution into two components, specific nucleotide stimulation, and cofactor requirements. *Biochemistry* 8 659–670 10.1021/bi00830a030 4893578

[B33] Smith C. S. Morrice N. A. Moorhead G. B. (2004). Lack of evidence for phosphorylation of *Arabidopsis thaliana* PII: implications for plastid carbon and nitrogen signaling. *Biochim. Biophys. Acta* 1699 145–154 10.1016/j.bbapap.2004.02.009 15158722

[B34] Strosser J. Ludke A. Schaffer S. Kramer R. Burkovski A. (2004). Regulation of GlnK activity: modification, membrane sequestration and proteolysis as regulatory principles in the network of nitrogen control in *Corynebacterium glutamicum*. *Mol. Microbiol.* 54 132–147 10.1111/j.1365-2958.2004.04247.x 15458411

[B35] Thomas G. Coutts G. Merrick M. (2000). The glnKamtB operon: a conserved gene pair in prokaryotes. *Trends Genet.* 16 11–14 10.1016/S0168-9525(99)01887-9 10637624

[B36] Truan D. Bjelic S. Li X. D. Winkler F. K. (2014). Structure and thermodynamics of effector molecule binding to the nitrogen signal transduction PII protein GlnZ from *Azospirillum brasilense*. *J. Mol. Biol.* 426 2783–2799 10.1016/j.jmb.2014.05.008 24846646

[B37] Truan D. Huergo L. F. Chubatsu L. S. Merrick M. Li X. D. Winkler F. K. (2010). A new PII protein structure identifies the 2-oxoglutarate binding site. *J. Mol. Biol.* 400 531–539 10.1016/j.jmb.2010.05.036 20493877

[B38] Zeth K. Fokina O. Forchhammer K. (2014). Structural basis and target-specific modulation of ADP sensing by the *Synechococcus elongatus* PII signaling protein. *J. Biol. Chem.* 289 8960–8972 10.1074/jbc.M113.536557 24519945 PMC3979405

[B39] Zhang Y. Pohlmann E. L. Serate J. Conrad M. C. Roberts G. P. (2010). Mutagenesis and functional characterization of the four domains of GlnD, a bifunctional nitrogen sensor protein. *J. Bacteriol.* 192 2711–2721 10.1128/JB.01674-09 20363937 PMC2876476

[B40] Zhang Y. Pu H. Wang Q. Cheng S. Zhao W. Zhang Y. (2007). PII is important in regulation of nitrogen metabolism but not required for heterocyst formation in the cyanobacterium *Anabaena* sp. *PCC* 7120. *J. Biol. Chem.* 282 33641–33648 10.1074/jbc.M706500200 17875643

[B41] Zhao M. X. Jiang Y. L. Xu B. Y. Chen Y. Zhang C. C. Zhou C. Z. (2010). Crystal structure of the cyanobacterial signal transduction protein PII in complex with PipX. *J. Mol. Biol.* 402 552–559 10.1016/j.jmb.2010.08.006 20708625

